# Supplementary material for: Low-Bias RNA Sequencing of the HIV-2 Genome from Blood Plasma
Source: J Virol. 2018 Dec 10;93(1):e00677-18. doi: 10.1128/JVI.00677-18 (PMC6288329; doi:10.1128/JVI.00677-18)
Supplement: Supplemental file 1 [file 88092048d7357d3ba8d680982f34463b_JVI.00677-18-s0001.pdf]

SUPPLEMENTAL MATERIAL FOR PUBLICATION

**Content**	**Page**
Supplementary Tables	2-5
Supplementary Figure legends	6
Supplementary Figures	7-13
Supplementary References	14

Supplementary Tables

Table S1. Clinical data and molecular properties of the analysed samples and sequences[1].

Sequence name	Accession number[1]	Age	Sex	Country	CD4 (cells/µl)	Viral load (cp/ml)	Sampling year	Clinical status at isolation	HIV-2 group	Predicted coreceptor tropism
TD031	TBA	60	F	Guinea-Bissau	407	107183	2010	Asymptomatic	A	R5
TD062	TBA	59	M	Guinea-Bissau	497	139519	2010	Asymptomatic	A	R5
A.GM.MCN13	AY509259	31	F	The Gambia	NA	NA	1988	Aymptomatic	A	R5X4
A.GM.MCR35	AY509260	31	F	The Gambia	NA	NA	1988	Aymptomatic	A	R5X4
A.IN.NNVA	EU980602	37	M	India	711	NA	2007	Asymptomatic	A	R5X4
A.SN.ST_JSP4_27	M31113	NA	F	Senegal	NA	NA	1986	Asymptomatic	A	R5
TD024	TBA	79	F	Guinea-Bissau	191	10560	2010	AIDS	A	R5
CBL20	TBA	28	M	The Gambia	18	NA	1988	AIDS	A	R5
RODR	TBA	32	M	Cape Verde	100	NA	1985	AIDS	A	R5X4
A.SN.ROD	BD413542	32	M	Cape Verde	100	NA	1985	AIDS	A	R5X4
A.CI.UC2	U389293	37	F	Ivory Coast	12	NA	1986	AIDS	A	R5X4
A.DE.BEN	M30502	42	M	Germany	56	NA	1987	AIDS	A	R5
A.GH.GH1	E02138	33	F	Ghana	NA	NA	<1988	AIDS	A	R5
A.GM.D194	A09995	NA	M	The Gambia	NA	NA	1987	AIDS	A	R5X4
A.GM.ISY_SBL	J04498	55	F	The Gambia	110	NA	1985	AIDS	A	R5X4
A.GW.CAM2CG	D00835	NA	NA	Guinea-Bissau	NA	NA	1987	AIDS	A	R5
A.GW.NIHZ	J03654	29	M	Guinea-Bissau	85	NA	1986	AIDS	A	R5X4
A.IN.CRIK_147	DQ307022	NA	M	India	NA	NA	<2005	AIDS	A	R5X4
A.PT.ALI	AF082339	NA	NA	Guinea-Bissau	NA	NA	NA	ARC	A	R5
A.DE.PEI2	U22047	NA	NA	Germany	NA	NA	NA	NA	A	R5X4
A.GW.MDS	Z48731	NA	NA	Guinea-Bissau	NA	NA	<1995	NA	A	R5
A.JP.NMC786_41	AB731742	NA	NA	Japan	NA	NA	2008	NA	A	R5X4
A.JP.NMC786_43	AB731743	NA	NA	Japan	NA	NA	2008	NA	A	R5X4
Average aymptomatic group					84	10560			A	40% R5X4
Average AIDS group					538	123351			A	50% R5X4

[1]Abbreviations: TBA = To be added; NA = Not available; F = Female; M = Male; AIDS = Acquired Immunodeficiency Syndrome; ARC = AIDS-related complex; R5 = CCR5 using virus; R5X4 = CCR5 and CXCR4 using virus; PNGS = potential N-linked glycosylations (Number of potential N-linked glycosylation sites (PNGS) as defined in N-GLYCOSITE(1)). Coreceptor tropism was assessed as described in (2). Net charge of sequences was determined based on each lysine and arginine contributing +1 and each aspartic acid and glutamic acid contributing -1. Total counts of amino acids were also assessed as described(3).

Table S1. Clinical data and molecular properties of the analysed samples and sequences (continued)[1].

Sequence name	Gag				Pol				Env			
	Length	Net charge	Total charge	PNGS	Length	Net charge	Total charge	PNGS	Length	Net charge	Total charge	PNGS
TD031	521	12	128	1	960	10	246	1	861	17	171	30
TD062	521	15	125	2	960	15	249	1	850	10	166	28
A.GM.MCN13	521	12	124	2	960	14	250	1	859	16	170	27
A.GM.MCR35	521	12	126	2	960	15	249	1	859	17	171	26
A.IN.NNVA	521	11	129	2	960	12	250	1	857	11	181	27
A.SN.ST_JSP4_27	521	13	131	1	960	12	244	1	860	18	176	30
TD024	521	12	128	2	954	12	248	1	856	14	172	31
CBL20	521	11	129	1	960	11	245	1	854	16	166	28
RODR	522	14	128	2	960	13	247	1	858	15	173	30
A.SN.ROD	522	14	128	1	951	11	247	1	858	15	173	30
A.CI.UC2	521	15	131	1	960	13	245	1	865	19	165	27
A.DE.BEN	521	16	134	1	960	13	241	1	860	21	177	25
A.GH.GH1	522	15	129	0	960	11	245	2	853	17	169	28
A.GM.D194	521	14	128	1	960	11	245	1	851	15	171	27
A.GM.ISY_SBL	520	10	130	2	960	13	245	1	847	6	186	25
A.GW.CAM2CG	521	11	127	2	960	14	248	1	856	17	169	29
A.GW.NIHZ	519	10	124	2	960	6	248	1	856	22	176	27
A.IN.CRIK_147	521	13	127	2	960	12	250	1	861	14	178	20
A.PT.ALI	521	9	129	1	960	14	246	1	862	19	179	26
A.DE.PEI2	521	15	125	1	960	11	249	1	858	6	170	25
A.GW.MDS	521	15	129	2	960	11	249	1	869	17	177	32
A.JP.NMC786_41	521	13	129	0	960	7	245	2	856	18	184	25
A.JP.NMC786_43	521	13	129	0	960	11	241	2	856	18	184	25
Average aymptomatic group	521	13	128	2	960	13	248	1	857	15	173	28
Average AIDS group	521	13	129	1	959	12	246	1	857	16	173	27

Table S1. Clinical data and molecular properties of the analysed samples and sequences (continued)[1].

Sequence name	Tat				Rev				Vif			
	Length	Net charge	Total charge	PNGS	Length	Net charge	Total charge	PNGS	Length	Net charge	Total charge	PNGS
TD031	130	8	40	1	100	4	30	0	215	16	52	0
TD062	130	8	38	0	100	5	25	0	215	18	60	0
A.GM.MCN13	130	10	38	0	107	5	31	0	215	12	52	0
A.GM.MCR35	130	9	37	0	107	4	32	0	215	11	53	0
A.IN.NNVA	130	1	41	0	100	3	27	0	215	16	54	1
A.SN.ST_JSP4_27	130	7	35	0	107	2	32	0	215	17	53	1
TD024	130	7	39	1	100	3	25	0	215	18	50	2
CBL20	130	8	34	1	103	4	28	0	215	13	53	0
RODR	130	9	39	1	100	2	28	0	215	16	58	0
A.SN.ROD	130	9	39	1	100	3	27	0	215	16	58	0
A.CI.UC2	130	9	35	0	103	1	33	0	215	16	54	0
A.DE.BEN	130	10	38	0	103	5	29	0	215	13	55	0
A.GH.GH1	130	8	34	0	103	2	32	0	215	16	56	0
A.GM.D194	130	8	36	0	103	4	30	0	215	14	52	0
A.GM.ISY_SBL	130	8	36	0	96	7	29	0	215	18	52	1
A.GW.CAM2CG	130	8	38	1	100	4	28	0	215	18	54	1
A.GW.NIHZ	130	7	41	0	107	3	29	0	215	16	56	0
A.IN.CRIK_147	130	3	35	0	100	3	25	0	215	11	55	0
A.PT.ALI	130	5	37	1	107	1	29	0	215	17	51	1
A.DE.PEI2	130	10	36	0	107	6	28	0	215	16	50	1
A.GW.MDS	130	6	38	0	100	5	27	0	215	16	54	0
A.JP.NMC786_41	130	10	38	0	96	4	32	0	215	16	54	0
A.JP.NMC786_43	130	8	38	0	96	4	32	0	215	14	56	0
Average aymptomatic group	130	7	38	0	103	4	29	0	215	16	54	0
Average AIDS group	130	8	37	0	102	3	29	0	215	16	54	0

Table S1. Clinical data and molecular properties of the analysed samples and sequences (continued)[1].

Sequence name	Vpr				Vpx				Nef			
	Length	Net charge	Total charge	PNGS	Length	Net charge	Total charge	PNGS	Length	Net charge	Total charge	PNGS
TD031	87	-2	28	0	112	2	24	1	257	-7	77	1
TD062	87	-3	25	0	112	1	27	0	257	-6	72	1
A.GM.MCN13	87	0	26	0	112	0	24	0	257	-9	79	1
A.GM.MCR35	87	0	26	0	112	0	24	0	215	-1	67	1
A.IN.NNVA	87	-2	28	0	112	0	22	0	257	-7	77	1
A.SN.ST_JSP4_27	87	0	26	0	112	1	23	0	256	-6	78	1
TD024	87	-1	27	0	112	0	22	0	257	-6	74	1
CBL20	87	-3	25	1	112	1	25	0	257	-5	75	1
RODR	87	-2	26	0	112	2	24	1	257	-10	76	2
A.SN.ROD	87	-2	26	0	112	2	24	1	257	-7	75	2
A.CI.UC2	68	5	19	0	112	1	25	0	257	-2	70	2
A.DE.BEN	87	-1	27	1	113	-4	30	0	257	-6	78	1
A.GH.GH1	87	0	26	1	112	1	25	0	256	-1	71	2
A.GM.D194	87	-1	27	0	112	1	25	0	257	-5	73	2
A.GM.ISY_SBL	87	-4	26	0	112	1	27	0	257	-11	77	1
A.GW.CAM2CG	87	-4	26	0	112	0	24	0	257	-7	77	2
A.GW.NIHZ	87	-3	27	0	112	-3	25	0	181	1	53	1
A.IN.CRIK_147	87	-5	27	0	112	0	22	0	172	0	52	0
A.PT.ALI	87	0	24	0	112	0	22	0	258	-5	71	1
A.DE.PEI2	87	-3	25	0	111	4	22	0	254	-5	67	1
A.GW.MDS	87	-4	26	0	112	1	25	0	256	-8	74	1
A.JP.NMC786_41	87	-1	25	0	112	-2	26	0	257	-9	73	1
A.JP.NMC786_43	87	-1	25	0	112	-1	25	0	257	-8	74	1
Average aymptomatic group	87	-1	27	0	112	1	24	0	257	-7	77	1
Average AIDS group	85	-2	26	0	112	0	25	0	257	-6	74	2

Supplementary Figure legends

Figure S1. Visualisation of the random hexamer bias.

Nucleotide composition per read position was assessed using FastQC for TD024 (A), TD031 (B), TD062 (C), CBL20 (D) for both the forward and reverse read mates.

Figure S2. Detailed molecular properties per gene.

Each HIV-2 gene was mapped according to positively charged amino acids are highlighted in blue (lysine [light blue] and arginine [dark blue]) and negatively charged amino acids in red (glutamic acid [light red] and aspartic acid [dark red]). Histidine positions are highlighted in pink. Potential N-linked glycosylation sites (PNGS) as defined in N-GLYCOSITE(1) are highlighted in black. Gap positions are shown in grey.

Supplementary Figures

Figure S1. Visualisation of the random hexamer bias.

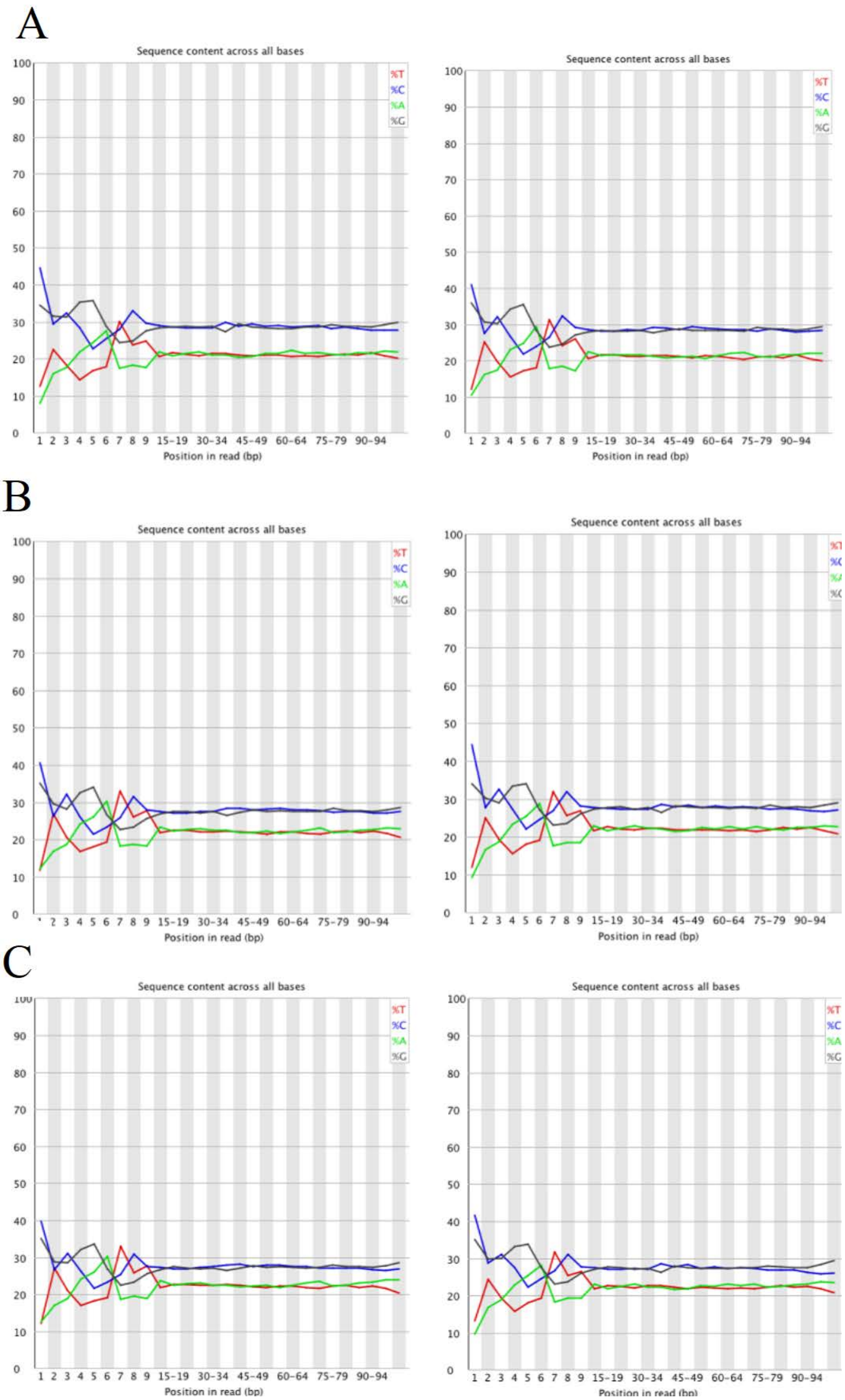

D

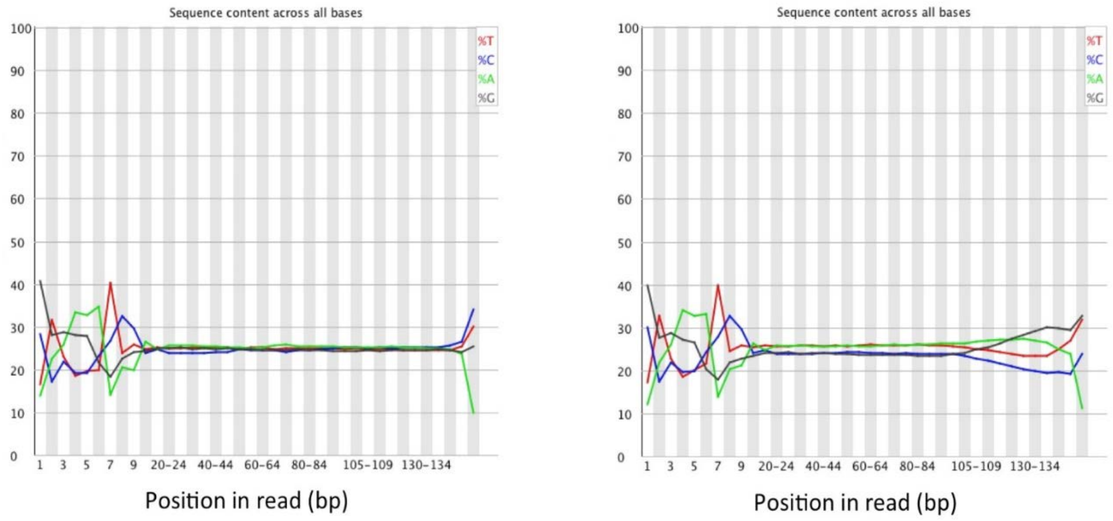
Sequence content across all bases
%T
%C
%A
%G
Position in read (bp)
Sequence content across all bases
%T
%C
%A
%G
Position in read (bp)

Figure S2. Detailed molecular properties per gene.

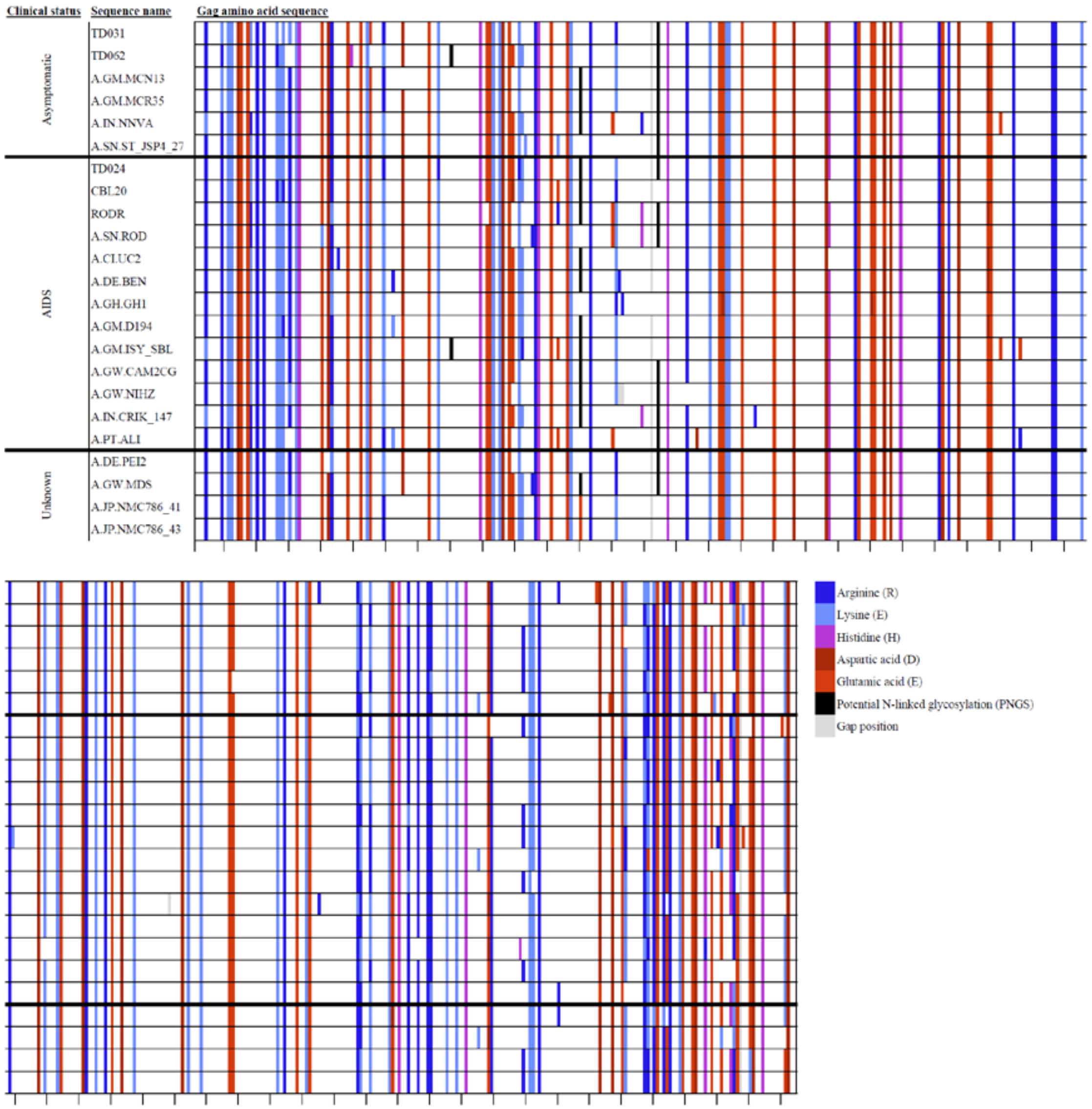

1
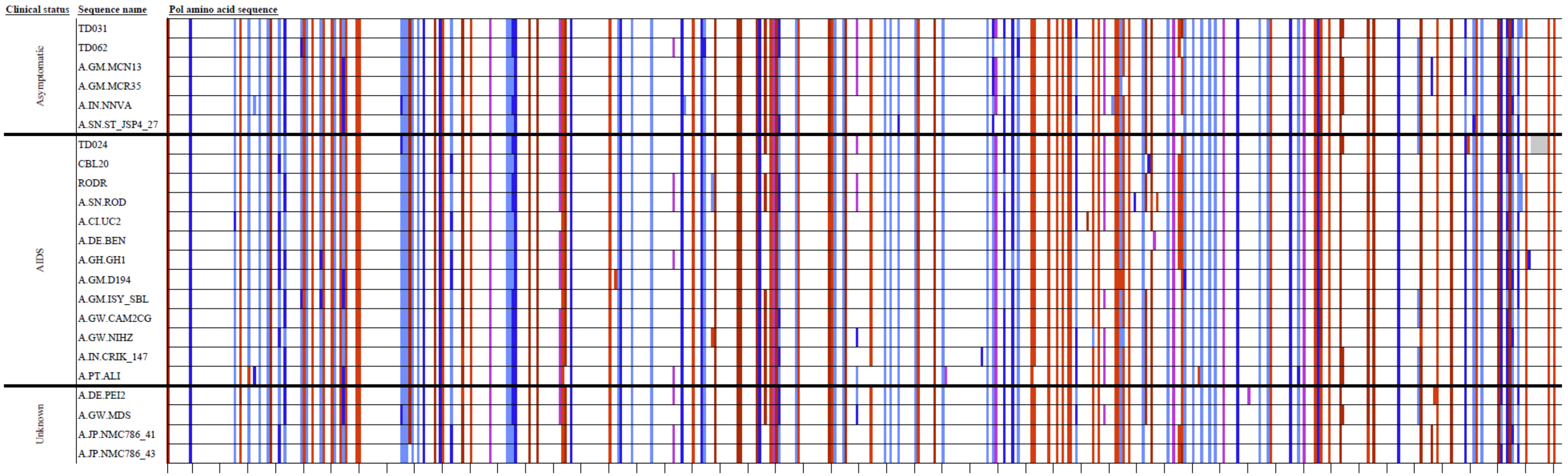
Clinical status
Sequence name
Pol amino acid sequence
Asymptomatic
AIDS
Unknown
TD031
TD062
A.GM.MCN13
A.GM.MCR35
A.IN.NNVA
A.SN.ST_JSP4_27
TD024
CBL20
RODR
A.SN.ROD
A.CI.UC2
A.DE.BEN
A.GH.GH1
A.GM.D194
A.GM.ISY_SBL
A.GW.CAM2CG
A.GW.NIHZ
A.IN.CRIK_147
A.PT.ALI
A.DE.PEI2
A.GW.MDS
A.JP.NMC786_41
A.JP.NMC786_43

2
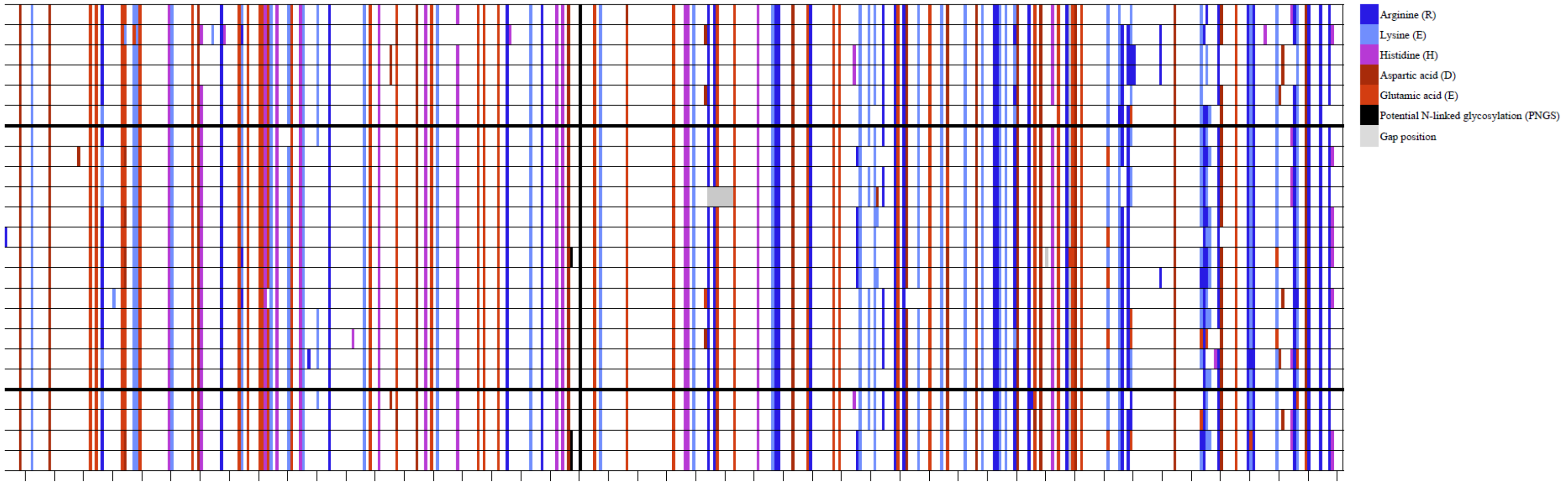
Arginine (R)
Lysine (E)
Histidine (H)
Aspartic acid (D)
Glutamic acid (E)
Potential N-linked glycosylation (PNGS)
Gap position

1

2

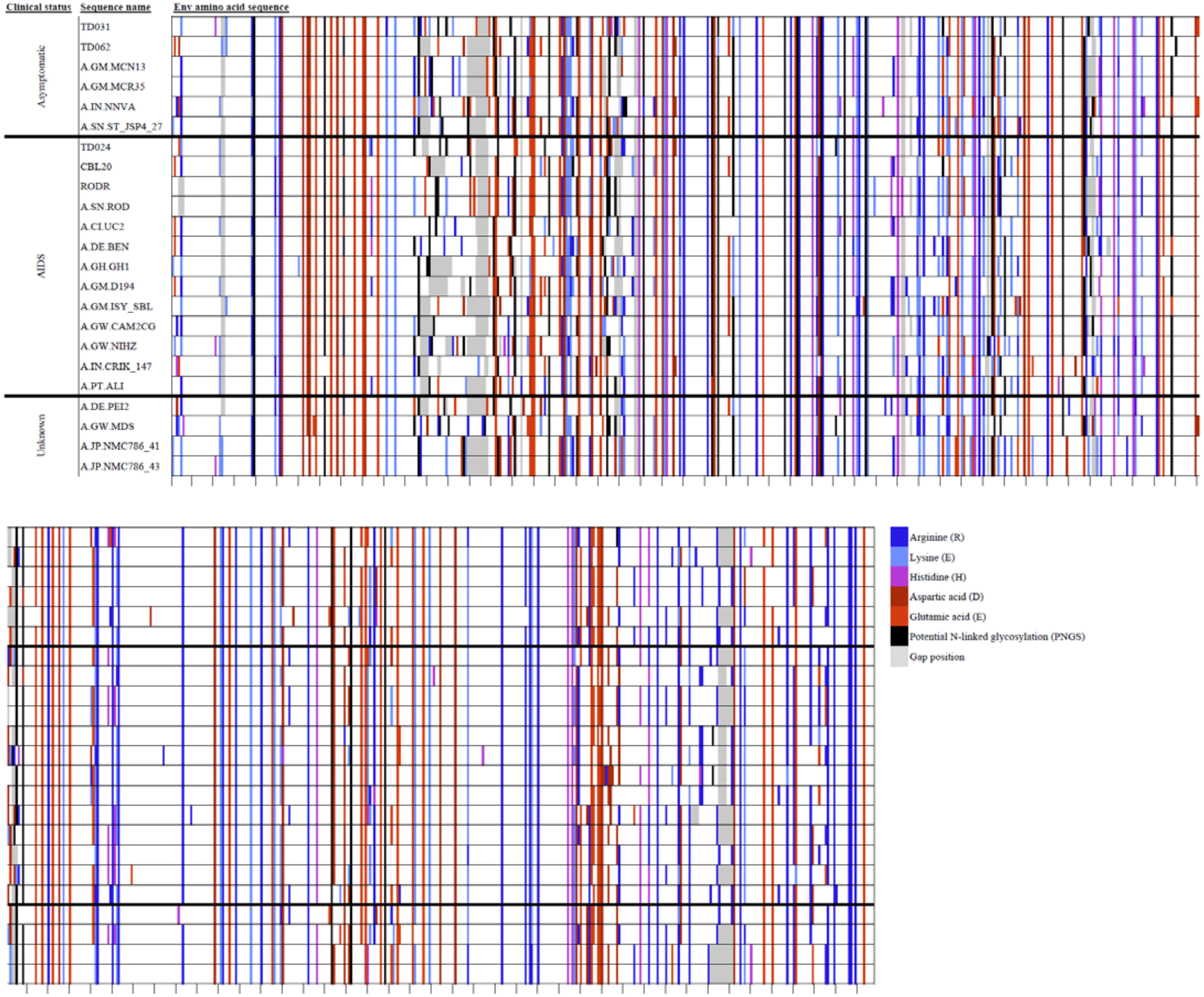
Clinical status
Sequence name
Env amino acid sequence
Asymptomatic
AIDS
Unknown
TD031
TD062
A.GM.MCN13
A.GM.MCR35
A.IN.NNVA
A.SN.ST_JSP4_27
TD024
CBL20
RODR
A.SN.ROD
A.CI.UC2
A.DE.BEN
A.GH.GH1
A.GM.D194
A.GM.ISY_SBL
A.GW.CAM2CG
A.GW.NIHZ
A.IN.CRIK_147
A.PT.ALI
A.DE.PEI2
A.GW.MDS
A.JP.NMC786_41
A.JP.NMC786_43
Arginine (R)
Lysine (E)
Histidine (H)
Aspartic acid (D)
Glutamic acid (E)
Potential N-linked glycosylation (PNGS)
Gap position

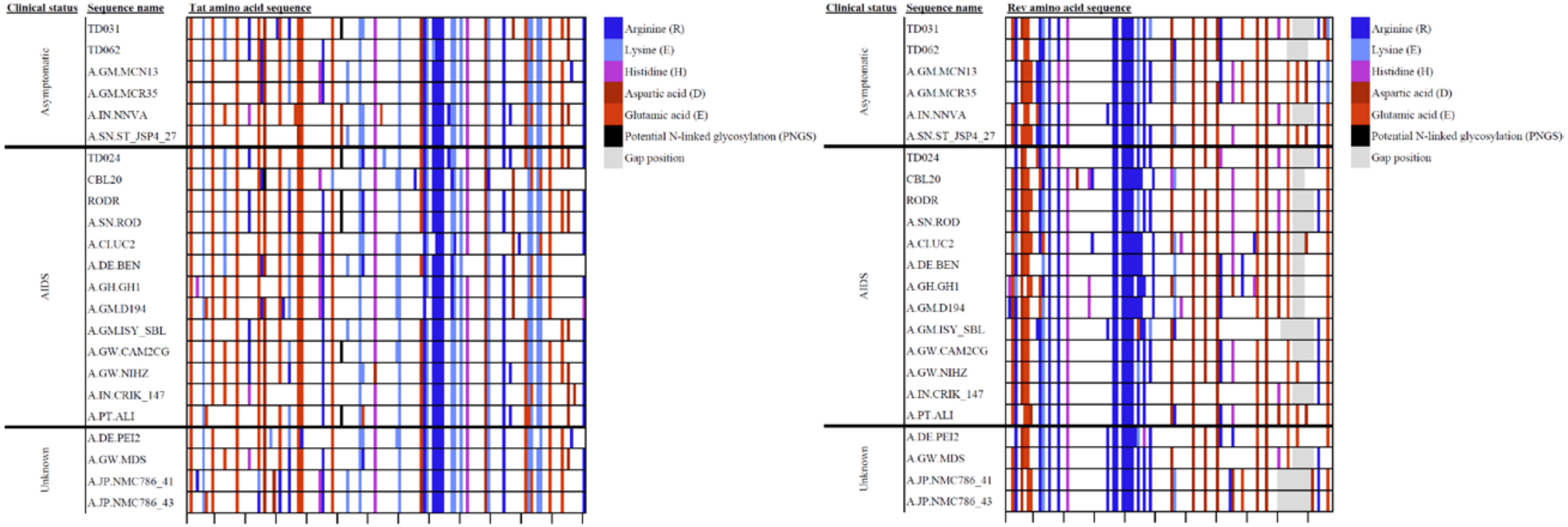
Clinical status
Sequence name
Tat amino acid sequence
Asymptomatic
AIDS
Unknown
TD031
TD062
A.GM.MCN13
A.GM.MCR35
A.IN.NNVA
A.SN.ST_JSP4_27
TD024
CBL20
RODR
A.SN.ROD
A.CI.UC2
A.DE.BEN
A.GH.GH1
A.GM.D194
A.GM.ISY_SBL
A.GW.CAM2CG
A.GW.NIHZ
A.IN.CRIK_147
A.PT.ALI
A.DE.PEI2
A.GW.MDS
A.JP.NMC786_41
A.JP.NMC786_43
Arginine (R)
Lysine (E)
Histidine (H)
Aspartic acid (D)
Glutamic acid (E)
Potential N-linked glycosylation (PNGS)
Gap position
Rev amino acid sequence

1

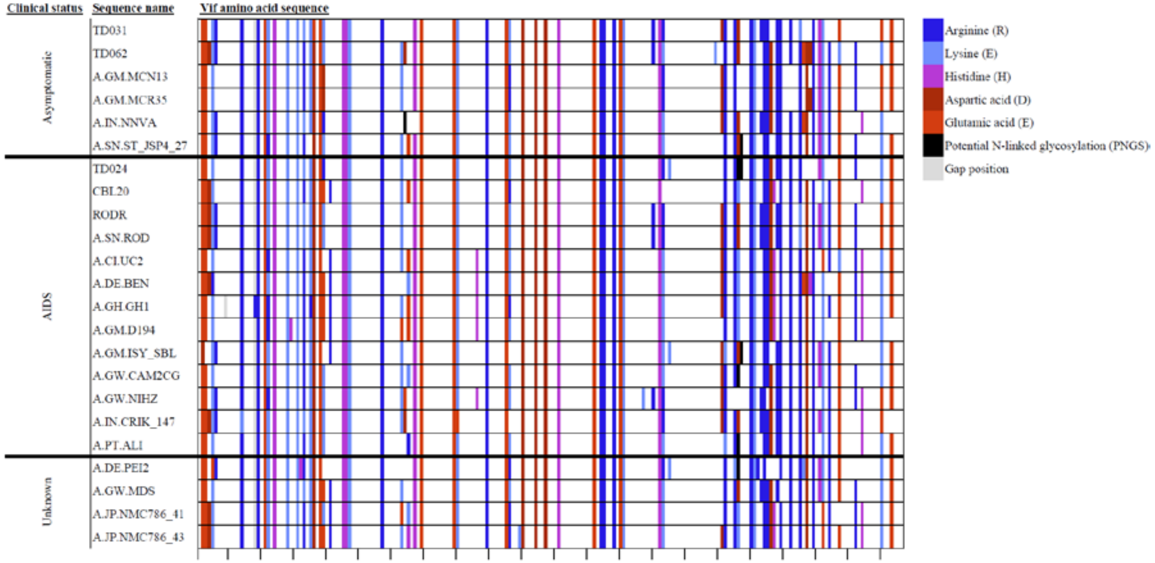
Clinical status
Sequence name
Vif amino acid sequence
Asymptomatic
AIDS
Unknown
TD031
TD062
A.GM.MCN13
A.GM.MCR35
A.IN.NNVA
A.SN.ST_JSP4_27
TD024
CBL20
RODR
A.SN.ROD
A.CI.UC2
A.DE.BEN
A.GH.GH1
A.GM.D194
A.GM.ISY_SBL
A.GW.CAM2CG
A.GW.NIHZ
A.IN.CRIK_147
A.PT.ALI
A.DE.PEI2
A.GW.MDS
A.JP.NMC786_41
A.JP.NMC786_43
Arginine (R)
Lysine (E)
Histidine (H)
Aspartic acid (D)
Glutamic acid (E)
Potential N-linked glycosylation (PNGS)
Gap position

2

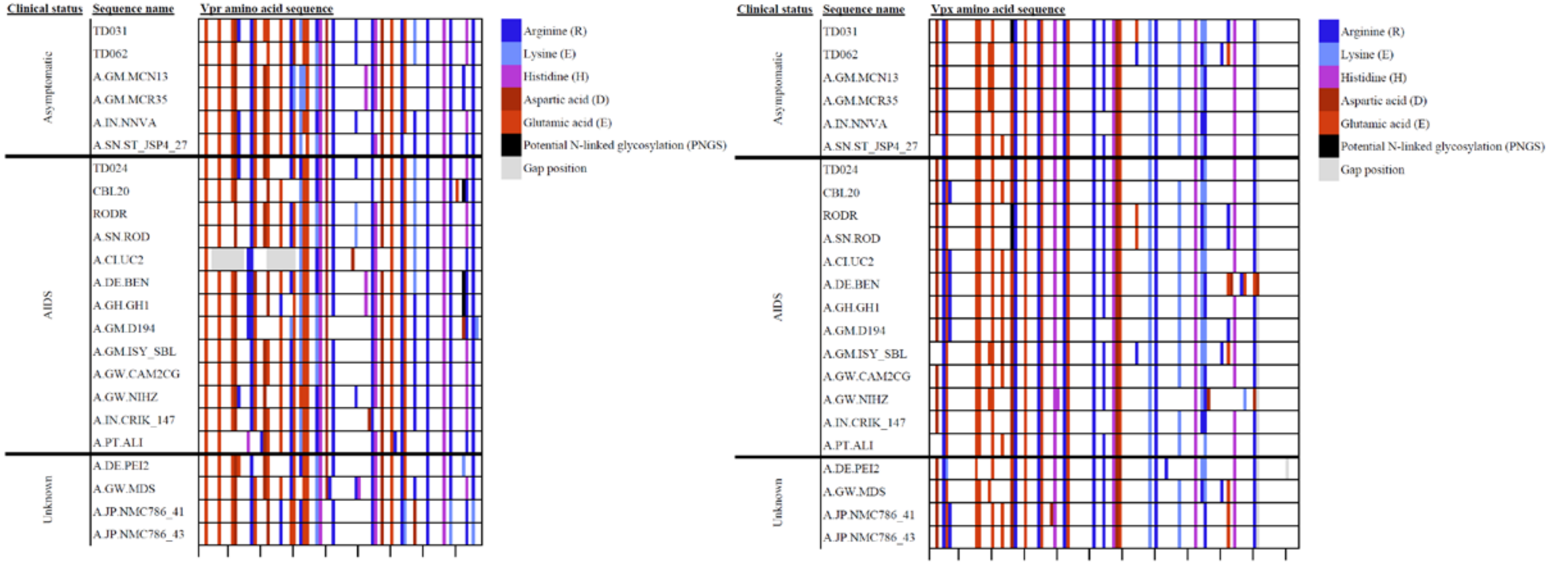
Clinical status
Sequence name
Vpr amino acid sequence
Asymptomatic
AIDS
Unknown
TD031
TD062
A.GM.MCN13
A.GM.MCR35
A.IN.NNVA
A.SN.ST_JSP4_27
TD024
CBL20
RODR
A.SN.ROD
A.CI.UC2
A.DE.BEN
A.GH.GH1
A.GM.D194
A.GM.ISY_SBL
A.GW.CAM2CG
A.GW.NIHZ
A.IN.CRIK_147
A.PT.ALI
A.DE.PEI2
A.GW.MDS
A.JP.NMC786_41
A.JP.NMC786_43
Arginine (R)
Lysine (E)
Histidine (H)
Aspartic acid (D)
Glutamic acid (E)
Potential N-linked glycosylation (PNGS)
Gap position
Clinical status
Sequence name
Vpx amino acid sequence
Asymptomatic
AIDS
Unknown
TD031
TD062
A.GM.MCN13
A.GM.MCR35
A.IN.NNVA
A.SN.ST_JSP4_27
TD024
CBL20
RODR
A.SN.ROD
A.CI.UC2
A.DE.BEN
A.GH.GH1
A.GM.D194
A.GM.ISY_SBL
A.GW.CAM2CG
A.GW.NIHZ
A.IN.CRIK_147
A.PT.ALI
A.DE.PEI2
A.GW.MDS
A.JP.NMC786_41
A.JP.NMC786_43
Arginine (R)
Lysine (E)
Histidine (H)
Aspartic acid (D)
Glutamic acid (E)
Potential N-linked glycosylation (PNGS)
Gap position

1

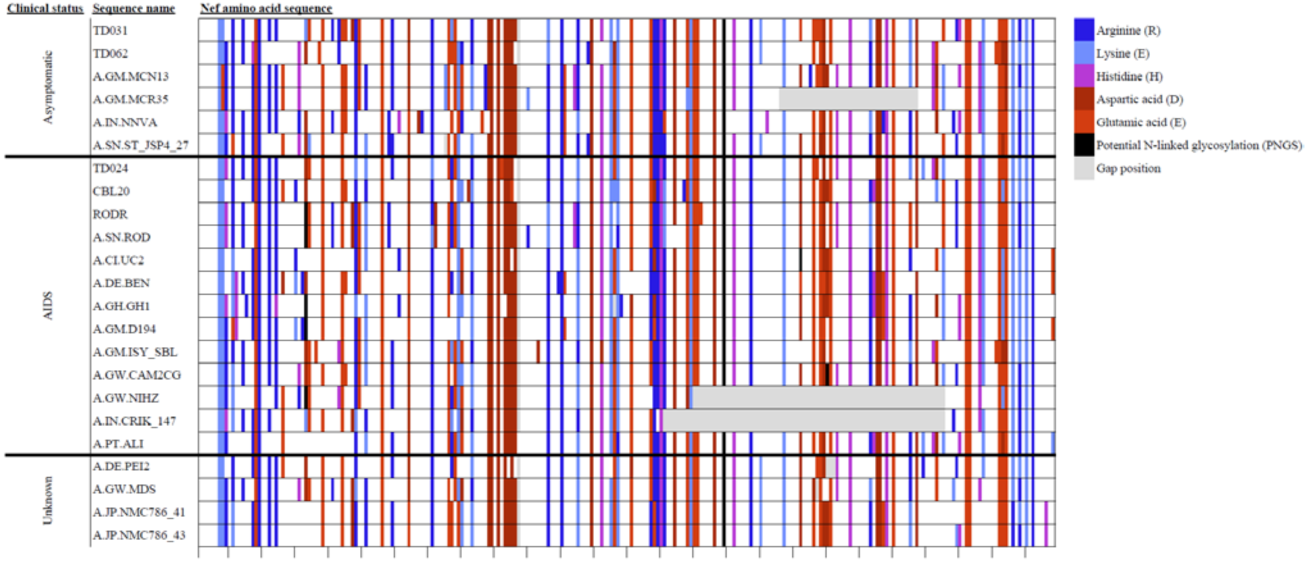
Clinical status
Sequence name
Nef amino acid sequence
Asymptomatic
AIDS
Unknown
TD031
TD062
A.GM.MCN13
A.GM.MCR35
A.IN.NNVA
A.SN.ST_JSP4_27
TD024
CBL20
RODR
A.SN.ROD
A.CI.UC2
A.DE.BEN
A.GH.GH1
A.GM.D194
A.GM.ISY_SBL
A.GW.CAM2CG
A.GW.NIHZ
A.IN.CRIK_147
A.PT.ALI
A.DE.PEI2
A.GW.MDS
A.JP.NMC786_41
A.JP.NMC786_43
Arginine (R)
Lysine (E)
Histidine (H)
Aspartic acid (D)
Glutamic acid (E)
Potential N-linked glycosylation (PNGS)
Gap position

2

Supplementary References

1. LANL. Los Alamos Sequence Database. www.hiv.lanl.gov. Accessed
2. Visseaux B, Hurtado-Nedelec M, Charpentier C, Collin G, Storto A, Matheron S, Larrouy L, Damond F, Brun-Vezinet F, Descamps D, Cohort ACH-. 2012. Molecular determinants of HIV-2 R5-X4 tropism in the V3 loop: development of a new genotypic tool. J Infect Dis 205:111-20.
3. Esbjornsson J, Mansson F, Martinez-Arias W, Vincic E, Biague AJ, da Silva ZJ, Fenyo EM, Norrgren H, Medstrand P. 2010. Frequent CXCR4 tropism of HIV-1 subtype A and CRF02_AG during late-stage disease--indication of an evolving epidemic in West Africa. Retrovirology 7:23.
